# Personal Agency Support Questionnaire in Acute Psychiatric Inpatients: Development and Instrument Validation Study

**DOI:** 10.2196/83366

**Published:** 2026-02-03

**Authors:** Tomomi Kajiwara, Yuta Hayashi, Masami Sugihara, Rie Chiba

**Affiliations:** 1 Department of Psychiatric and Mental Health Nursing, Graduate School of Nursing, Mukogawa Women’s University, Nishinomiya, Japan; 2 Department of Public Health, Graduate School of Health Sciences, Kobe University, Kobe, Japan; 3 Department of Nursing, Graduate School of Health Science, Kobe University, Kobe, Japan; 4 Department of Nursing, Sawa Hospital, Social Medical Corporation Hokutokai, Toyonaka, Japan; 5 Human Health Sciences, Graduate School of Medicine, Kyoto University, 53 Shogoinkawaharacho, Sakyo-ku, Kyoto, 606-8507, Japan, 81 75-751-3919

**Keywords:** inpatients, mental disorders, mental health, personal autonomy, psychometrics, surveys and questionnaires

## Abstract

**Background:**

Promoting personal agency may reduce perceived coercion and facilitate recovery in acute psychiatric care. However, no patient-reported tool currently exists to evaluate support for personal agency in this setting.

**Objective:**

This study aimed to develop a patient-reported tool (the Personal Agency Support Questionnaire [PASQ]) to assess perceived support for personal agency and to evaluate its psychometric properties among inpatients in acute psychiatric wards.

**Methods:**

We used a literature review and focus group interviews to generate a pool of items for the questionnaire, which was then refined using cognitive interviews and a pretest. We evaluated the construct validity, internal consistency, and test-retest reliability of the newly developed PASQ using a cross-sectional survey of inpatients in acute psychiatric wards. This study was conducted in collaboration with individuals who have lived experiences of mental illness.

**Results:**

We analyzed data from 109 respondents (response rate: 109/178, 61.2%; mean age: 52.9, SD 16.9 years; women participants: 59/109, 54.1%; diagnosed with schizophrenia: 61/109, 56%). The 10-item PASQ demonstrated excellent convergent validity and acceptable discriminant validity. Internal consistency was high (Cronbach α=0.92), and test-retest reliability was moderate (intraclass correlation coefficient 0.68).

**Conclusions:**

This PASQ is a valuable tool for assessing personal agency support in acute psychiatric wards, demonstrating promise for both clinical use in acute psychiatric wards and clinical research.

## Introduction

Acute psychiatric wards provide rapid treatment to stabilize the symptoms of individuals whose acute mental health challenges cannot be fully managed by community services [1]. Because acute psychiatric symptoms temporarily influence cognitive processes and impair behavioral control [2,3], involuntary treatment and behavioral restrictions are sometimes used to ensure safety [4]. Although many inpatients experience therapeutic benefits from psychiatric hospitalization, it is often a negative experience, with previous studies indicating a high frequency of traumatic events [5,6] that affect patients’ well-being and self-worth [7,8]. This increased distress caused by inpatient stays can lead to the subsequent avoidance of mental health services [6].

The psychiatric care system in Japan has one of the largest numbers of psychiatric beds globally (approximately 319,000), and a long average length of stay (263.2 d) remains a persistent concern [9]. To address this issue, the government has been promoting a shift from long-term hospitalization to community-based care, making the role of acute psychiatric wards—which emphasize short-term stabilization of acute symptoms—increasingly important [10]. Almost half of patients occupying these beds are there involuntarily, and Japanese acute wards use restrictive treatments at significantly higher rates than other countries [11,12]. Key strategies for addressing this urgent issue include minimizing the use of restrictive practices and enhancing staff awareness of the importance of supporting patients’ personal agency [5].

Personal agency is defined as an individual’s ability to control their own lives, pursue their goals [13,14], and perceive a sense of ownership of their own behavior [15]. It incorporates concepts such as intentionality, forethought, self-reactivity, and self-reflectiveness and operates through cognitive, motivational, affective, and choice processes [13]. Personal agency has been conceptualized as encompassing both intrinsic (internal states of being, such as self-confidence and self-awareness) and instrumental (ways of acting, such as goal-directed decision-making and behavioral control) agency [16,17]. This framework builds upon prior conceptualizations of agency, such as Kabeer’s distinction between “power within” and “power to” [18].

Personal agency is conceptually related to other key concepts in psychiatric care, such as autonomy, empowerment, and personal recovery. Autonomy is commonly understood as acting in line with one’s own values in psychiatry [19] and is regarded as an outcome achieved through processes that constitute personal agency [13,19,20]. In mental health services, support for autonomy emphasizes the promotion of self-determination, with shared decision-making highlighted as a contemporary approach [21,22]. Empowerment is a process by which individuals gain greater control over the decisions and actions that affect their health [23], encompassing both personal agency and broader social and environmental transformation [24]. Personal recovery refers to living a fulfilling life despite experiencing psychiatric symptoms [25] and represents a comprehensive framework in which personal agency is a central driving force that facilitates the recovery process [26-28]. In acute psychiatric care, symptoms and safety requirements often limit the extent to which higher-order processes, such as autonomy and empowerment, can be expressed [19,29]. In contrast, personal agency remains a central mechanism within the recovery process regardless of symptom severity or treatment phase [26-28,30]. Therefore, clarifying how personal agency is supported in daily care is particularly important in acute settings. Personal agency plays a crucial role in reducing feelings of coercion and involuntariness during hospitalization [8]; moreover, it contributes to patient engagement in care and long-term recovery [31,32].

Despite its importance, there are currently no tools available to assess support for personal agency from the patient’s perspective. In acute settings, understanding how patients perceive the support they receive and how their voice is reflected in clinical practice is essential for maintaining a balance between safety and patients’ personal agency and ultimately fostering care that supports their recovery. Therefore, the aim of this study was to develop and validate the Personal Agency Support Questionnaire (PASQ) as a practical checklist-type questionnaire, rather than a psychometric scale that measures latent constructs, that captures patient-perceived support for personal agency in acute psychiatric settings.

## Methods

### Overall Design

This study was conducted within acute psychiatric care settings, where supporting personal agency is especially relevant and challenging owing to acute symptoms, rapid clinical decision-making, and safety needs. It consisted of 3 phases. The development and psychometric evaluation of the PASQ were guided by the COSMIN (Consensus-Based Standards for the Selection of Health Measurement Instruments) checklist, which provides international standards for studies on measurement properties of health-related instruments (Checklist 1).

In phase 1, we conducted a literature review and focus group interviews to generate an item pool for the new questionnaire. Subsequently, we conducted cognitive interviews to refine these items.

In phase 2, we conducted a pretest to verify whether the questionnaire items were appropriate for the target group and evaluate the likely responses.

In phase 3, we evaluated the validity and reliability of the PASQ by a cross-sectional survey of inpatients in acute psychiatric wards. Reporting of phase 3 followed the STROBE (Strengthening the Reporting of Observational Studies in Epidemiology) statement for observational studies [33] to ensure methodological rigor and transparent reporting (Checklist 1).

### Phase 1: Developing an Initial Item Pool and Cognitive Interview

In phase 1, we conducted a literature review and focus group interviews guided by best-practice recommendations for item pool generation [34]. We referred to existing patient-reported scales assessing various factors, such as recovery, empowerment, and autonomy, that had demonstrated reliability and validity for evaluating individuals with mental illness. From these scales, we incorporated the perspectives of research collaborators with lived experience of mental illness and extracted items according to 3 criteria: (1) the support aligned with personal agency; (2) the support included only the direct experiences of patients’ interactions with staff, rather than support provided by hospitals or services; (3) the support focused on foundational aspects of personal agency relevant to acute psychiatric care, rather than directly targeting long-term social participation or self-actualization.

Through team discussions, we grouped items that met these criteria according to similarity in meaning to reduce redundancy and organize the item pool.

In parallel, focus group interviews were conducted with individuals who had experienced acute psychiatric hospitalization within the past 10 years. Participants were recruited from welfare service centers and nonprofit organizations in the Kansai region of Japan using convenience sampling (this approach was used primarily because of existing collaborative networks and logistical feasibility). The focus group interviews aimed to identify support that contributed to personal agency during acute hospitalization. Data collection and analysis were conducted iteratively and concurrently to capture diverse narratives, including unspoken perspectives and differing patterns. For the analysis, we focused on the experiences that were commonly identified as support for personal agency across participants, taking into account differences in backgrounds and experiences. These experiences were integrated and organized according to similarity.

Based on these findings, semantically redundant items were removed or integrated. Through collaborative discussions with our research collaborators with lived experience of mental illness, we selected items that supported personal agency while also considering the psychological burdens and contextual constraints of acute psychiatric settings. During this process, we examined how each support type was perceived and its effectiveness in acute contexts. For example, for the item of “information provision,” participants stated that “too much information can be confusing, and it is more important to understand what is happening now and what to do next.” Upon reflection of such views, the item was refined to ensure that it evaluated whether information was provided at a time and in a manner that patients could understand and accept.

Content validity was examined collaboratively alongside four research collaborators with lived experience of mental illness and one clinical expert. All reviewed each item for clarity, relevance to support for personal agency, and appropriateness for acute psychiatric contexts. A cognitive interview was then conducted with one of the focus groups to confirm that the instructions and item wording could easily be understood.

### Phase 2: Pretest

In phase 2, we conducted a pretest with 20 people from 2 acute wards of a psychiatric hospital. One of these wards later participated in the main survey in phase 3 in May 2024. The pretest involved assessing the extent of the burden on questionnaire respondents and identifying items that were frequently left unanswered or often received identical responses.

### Phase 3: Evaluation of the Questionnaire

#### Setting and Participants

To evaluate the validity and reliability of the PASQ, we conducted a cross-sectional survey among inpatients in 6 acute psychiatric wards across 2 psychiatric hospitals in Japan. The hospitals were selected using convenience sampling. Coincidentally, both hospitals were accredited by the Japan Council for Quality Health Care, which ensured a more structured and higher-quality treatment environment compared with many other psychiatric facilities in Japan.

Inpatients were included in this study if they met the following criteria: (1) they had been hospitalized for at least 1 week during their current stay; (2) the attending psychiatrist had confirmed that their mental state and treatment would not be affected by participation in the research; and (3) they were aged 18 years or older. We excluded inpatients who (1) were in isolation or physically restrained; (2) were primarily being treated for a physical illness; and (3) had a diagnosis of dementia or intellectual disability. Nursing managers in each ward identified eligible patients admitted during the study period (from May to September 2024). The first author explained the study to each participant and obtained their informed consent. The estimated required sample size for phase 3 was 100 participants, in accordance with the COSMIN (Consensus-Based Standards for the Selection of Health Measurement Instruments) checklist [35], which provides international guidelines for measurement studies.

#### Data Collection Procedures

The first author distributed paper-based self-administered questionnaires to each participant. If requested, the first author read the questions aloud or completed responses on the participants’ behalf, using only the exact wording of the items without providing additional explanations to minimize response bias. Participants were informed that their responses would remain confidential, would not affect their treatment, and would never be disclosed to the hospital staff involved in their care. They could either return the questionnaire to the first author directly or seal it in an envelope and place it in the ward’s collection box to ensure privacy.

#### Measures

##### The Personal Agency Support Questionnaire

The PASQ is a newly developed tool for assessing perceived support for personal agency even under restricted environments, including acute psychiatric care settings. It is rated on a 5-point Likert-type scale (0=“not at all” to 4=“very much”). A higher score indicates higher perception of the support for personal agency. As it assesses it in a checklist-like manner, it is not expected to be normally distributed. The Japanese version of the scale is provided in Multimedia Appendix 1.

##### The Japanese Version of Brief INSPIRE (Brief INSPIRE-J)

Given that personal agency is considered a central mechanism supporting personal recovery, and recovery-oriented support is conceptually linked to personal agency, we used the Brief INSPIRE-J to assess convergent validity. It is a shortened, 5-item version of the INSPIRE measure. Each item corresponds to 1 aspect of the concept of personal recovery (connection, hope, identity, meaning and purpose, and empowerment) and is rated on a 5-point Likert-type scale (0=“not at all” to 4=“very much”) [36]. A higher score indicates greater satisfaction of the service user with the professional support they received in their recovery-oriented practice. The validity and reliability of Brief INSPIRE-J have been confirmed in Japan (Cronbach *α*≥0.82) [37]. In this study, the Brief INSPIRE-J exhibited high internal consistency, with a Cronbach α of 0.92.

##### The Japanese Version of the Kessler 6-Item Psychological Distress Scale

Because the psychological distress scale assesses internal emotional states [38], while the PASQ measures the perception of support, these 2 scales are theoretically assumed to assess conceptually distinct constructs. Therefore, we used the Kessler 6-item Psychological Distress Scale (K6) to examine divergent validity. The K6 comprises 6 items that ask respondents how frequently they have experienced symptoms of psychological distress. It is rated on a 5-point Likert-type scale (0=none of the time to 4=all the time) [38], with higher scores indicating a greater likelihood of the respondent having experienced distress. The Japanese version demonstrated equivalent screening performance to that reported for the original English versions (the area under the receiver operating characteristic curve 0.94, 95% CI 0.88‐0.99) [39]. For K6 in this study, Cronbach *α*=0.80, indicating good internal consistency.

### Statistical Analysis

First, we conducted a descriptive analysis to examine the distribution of responses. Convergent validity was assessed assuming a significant and positive correlation with Brief INSPIRE-J; divergent validity was examined assuming an insignificant correlation with K6. The Shapiro-Wilk test demonstrated that the PASQ had a nonnormal distribution; therefore, Spearman’s rank correlation coefficients, 95% CI, and *P* values were calculated. Correlation coefficients were interpreted based on Akoglu [40] as follows: 0.00‐0.19: none/very weak; 0.20‐0.39: weak; 0.40‐0.59: moderate; 0.60‐0.79: strong; and 0.80‐1.00: very strong.

We calculated Cronbach α coefficients to measure internal consistency, with a value of 0.70 or higher considered sufficient [41]. We also calculated Cronbach α after removing each item and item-to-total correlations, which indicated how well each item aligned with the overall construct. In addition, to prevent inflation of the correlation value, we calculated corrected interitem correlations and the correlation between each item and the sum of the others. Item-to-total correlation and corrected interitem correlations of 0.30 or higher were considered acceptable [42], suggesting that the item contributed well to the overall reliability of the PASQ.

We assessed test-retest reliability in targeting 20 participants over approximately 2 weeks. This interval was established based on previous research, assuming that the memory and experience of the first response would not influence the second response and that little would change between responses. We calculated the intraclass correlation coefficients (ICCs) between the 2 time points to examine test-retest reliability. ICC values were classified as poor (<0.50), moderate (0.50‐0.75), good (0.75‐0.90), or excellent (>0.90), in line with Koo and Li [43]. Because the PASQ was developed as a short assessment questionnaire in a checklist manner, following previous studies [44,45], we did not perform factor analysis.

All statistical analyses were performed in *R* (Version 4.4.1; R Core Team) [46]. We used the psych [47] and tidyverse [48] packages for descriptive statistics and reliability analyses and the psych [47], lavaan [49], and irr [50] packages for ICCs. A *P* value of <.05 was considered statistically significant (2-tailed test).

### Patient and Public Involvement

Because this research focused on patients’ subjective experiences, 4 individuals who had experienced acute psychiatric admissions were involved as research collaborators throughout this study. They advised on the clarity and comprehensibility of item wording and the survey procedures to ensure that the items reflected patients’ lived experiences during acute psychiatric hospitalization. Their feedback led to some of the items being revised to better reflect perceived support that is responsive to the individual’s sense of readiness and current mental state. This patient and public involvement process followed ethical guidelines and adhered to the principles of meaningful involvement [51]. The study collaborators received a 5000 Japanese yen (US $31.60) gift card as a token of appreciation for attending 2 or 3 meetings.

### Ethical Considerations

Because this study involved acute psychiatric inpatients, certain considerations were applied to the recruitment of participants. Attending psychiatrists could restrict the first author from approaching patients to explain the study, if such contact could negatively impact patients’ treatment. The first author, who had no clinical or organizational role at the hospitals, explained the study to individual patients privately, without staff present. The voluntary nature of participation, the absence of any effect on treatment, and confidentiality were explained both verbally and in writing, and written informed consent was obtained from all patients. To ensure privacy, data were anonymized in a linkable manner to allow for consent withdrawal. Personal identifiers were stored separately. Data were managed on a password-protected computer and in a locked cabinet. No compensation was provided to participants. The study was approved by the Ethics Committee of the Kobe University Graduate School of Health Sciences and the Graduate School of Medicine at Kyoto University (approval 1172 and R4367), as well as the Ethics Committee of the participating hospital (approval 2024‐1).

## Results

### Phase 1: Developing an Initial Item Pool and Cognitive Interview

From the 330 items extracted from existing patient-reported measures, 94 items were selected according to item-selection criteria and grouped according to similarities (eg, respect for strengths and values, support for coping with difficulties, and assistance with decision-making). Focus group interviews were conducted with 12 individuals. Over 8 sessions, commonly reported experiences of support for personal agency in acute settings were identified, such as feeling safe, the ability to express thoughts openly, and the ability to make choices based on recovery level. By integrating insights from both the literature review and the focus group interviews, 10 items were selected, and consensus was reached among the researchers and research collaborators on all items, including their wording and clarity. The cognitive interview confirmed that all items could be easily understood by patients. The instructional texts were revised to ask respondents to reflect on the support received throughout their entire hospitalization and to rate support provided by all staff members in the ward rather than specific individuals.

### Phase 2: Pretest

The pretest was conducted with 21 participants from 2 wards. Although 4 participants required assistance (eg, reading support), all completed the questionnaire within approximately 5 minutes. No missing data or extreme response biases were observed.

### Phase 3: Evaluation of the Questionnaire

#### Study Participants

During the recruitment period, we explained the study to 113 of 178 eligible inpatients. Four participants did not return their questionnaires, but the remaining 109 (response rate 61.2%) provided responses and were included in the analysis. Of these 109 participants, 14 (12.8%) requested assistance with either reading the questions or writing their answers. There were no missing values for the main variables. Participant characteristics are presented in Table 1. Over half of the participants (59/109, 54.1%) were women. The overall mean age of participants was 52.9 (SD 16.9) years. Schizophrenia was the most common diagnosis (62/109, 56%), followed by mood disorders (32/109, 29.4%).

**Table 1.** Demographic and clinical characteristics of respondents^a^.

Variables	Values (N=109)
Age (y), mean (SD)	52.9 (16.9)
Duration of illness (y), mean (SD)	18.9 (15.9)
Length of current hospitalization (d), mean (SD)	36.4 (30.9)
Gender, n (%)	
Women	59 (54.1)
Men	50 (45.9)
Diagnosis, n (%)	
Schizophrenia	61 (56.0)
Mood disorders	32 (29.4)
Substance abuse	7 (6.4)
Anxiety disorder	4 (3.7)
Developmental disability	4 (3.7)
Epileptic psychosis	1 (0.9)
Type of hospitalization at the time of admission, n (%)	
Involuntary hospitalization for medical protection	66 (60.6)
Voluntary hospitalization	34 (31.2)
Involuntary hospitalization	7 (6.4)
Emergency hospitalization	2 (1.8)
Type of hospitalization at the time of the survey, n (%)	
Involuntary hospitalization for medical protection	56 (51.4)
Voluntary hospitalization	49 (45.0)
Involuntary hospitalization	4 (3.7)
Number of previous admissions to a psychiatric hospital, n (%)	
None	24 (22.9)
One	10 (9.5)
Two or more	71 (67.6)

a Subsample sizes vary for some variables because of missing responses (n=107 for age, n=105 for number of previous admissions to a psychiatric hospital, and n=99 for duration of illness).

#### Distribution of the Responses

**Figure 1.**
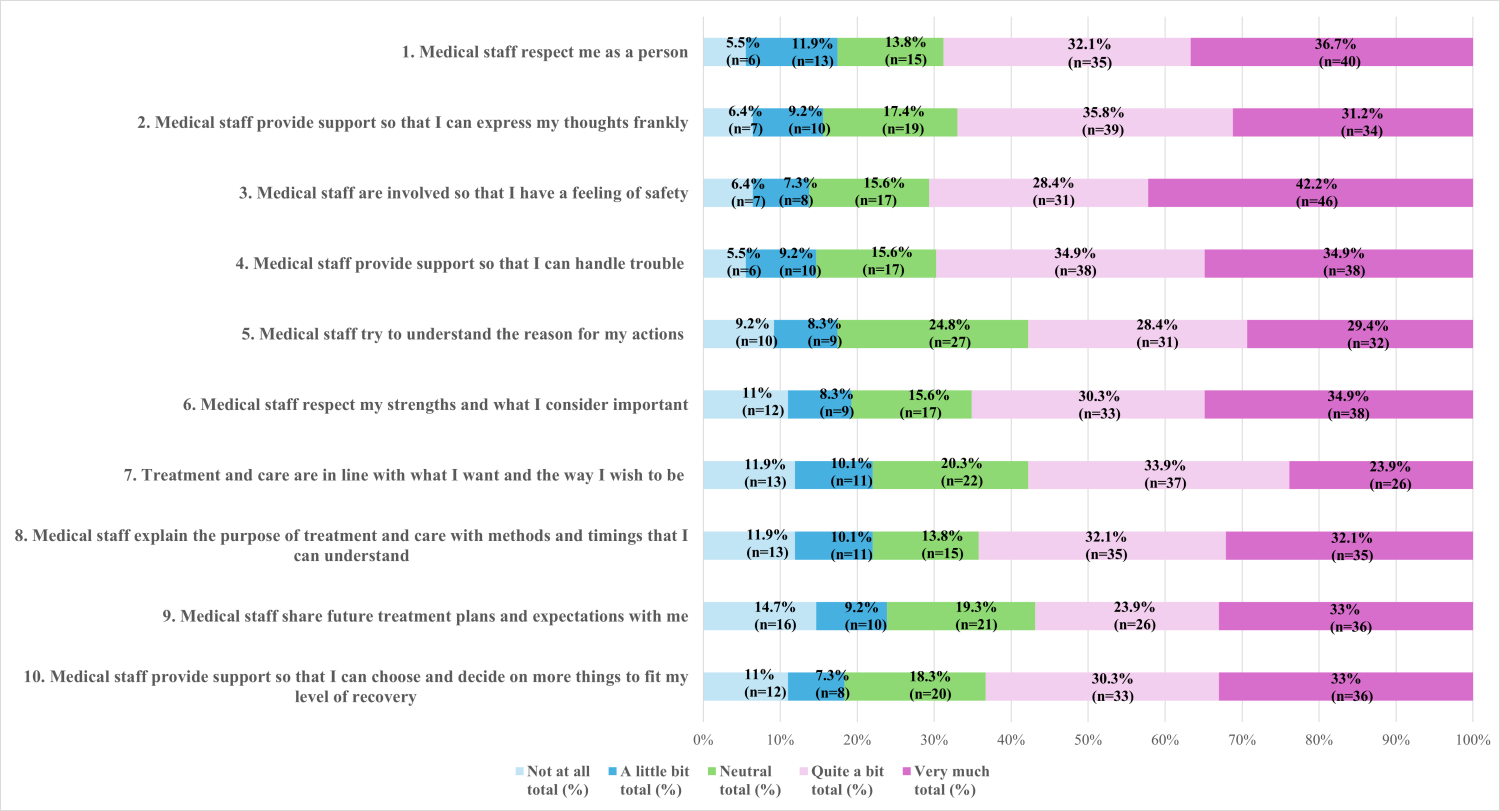
Distribution of responses to the Personal Agency Support Questionnaire items (N=109). Bars represent the percentage of participants selecting each response option on a 5-point Likert scale: not at all, a little bit, neutral, quite a bit, and very much.

Table 2 and Figure 1 illustrate the distribution of the responses to each item of the PASQ. Responses were generally skewed toward the higher categories (“quite a bit” and “very much”), with a median score of 3 for all items. Mean scores ranged from 2.48 (SD 1.29) to 2.93 (SD 1.21). A high proportion of respondents (almost 70%) answered “quite a bit” or “very much” to the following items: “Medical staff respect me as a person (75/109, 68.8%),” “Medical staff are involved so that I have a feeling of safety (77/109, 70.6%),” and “Medical staff provide support so that I can handle trouble (76/109, 69.7%).” Although these 3 items and item 6 demonstrated ceiling effects, they were retained to maintain content validity. In contrast, less than 60% of participants answered “quite a bit” or “very much” to the following items: “Medical staff try to understand the reason for my actions (63/109, 57.8%),” “Treatment and care are in line with what I want and the way I wish to be (63/109, 57.8%),” and “Medical staff share future treatment plans and expectations with me (62/109, 56.9%).”

**Table 2.** Personal Agency Support Questionnaire item responses and item-to-total correlations (N=109).

Item^a^	Mean (SD)	Median (IQR^b^; range)	Cronbach α when item is deleted^c^	Item-to-total correlation^d^	Corrected Interitem correlation^e^
1	2.83 (1.21)	3 (2; 0-4)	0.92	0.65	0.57
2	2.76 (1.18)	3 (2; 0-4)	0.91	0.78	0.72
3	2.93 (1.21)	3 (2; 0-4)	0.91	0.71	0.64
4	2.84 (1.16)	3 (2; 0-4)	0.91	0.81	0.76
5	2.61 (1.25)	3 (2; 0-4)	0.92	0.63	0.54
6	2.70 (1.32)	3 (2; 0-4)	0.91	0.75	0.67
7	2.48 (1.29)	3 (1; 0-4)	0.91	0.79	0.73
8	2.62 (1.35)	3 (2; 0-4)	0.91	0.80	0.74
9	2.51 (1.41)	3 (2; 0-4)	0.91	0.81	0.75
10	2.67 (1.31)	3 (2; 0-4)	0.90	0.84	0.79

a The wording of each item is presented in Figure 1.

b IQR: interquartile range.

c Cronbach α when item is deleted: reliability coefficient of the scale if the respective item is removed.

d Item-to-total correlation: correlation between the item and the total scale score excluding that item.

e Corrected interitem correlation: average correlation between the item and all other items.

#### Convergent and Divergent Validity

We found significant positive correlations between the total PASQ and total Brief INSPIRE-J scores (ρ=0.75, 95% CI 0.63‐0.85; *P*<.001). There were significant weak and negative correlations between the total PASQ and total K6 scores (ρ=−0.32, 95% CI −0.49 to −0.13; *P*<.001).

#### Reliability

The total score of the PASQ demonstrated sufficient homogeneity of all items, with a Cronbach α coefficient of 0.92. No single item substantially reduced the internal consistency, with Cronbach α coefficients ranging from 0.90 to 0.92 when each item was removed. Item-to-total correlations ranged from 0.63 to 0.84, indicating strong associations between each item and the overall scale, while corrected interitem correlations ranged from 0.54 to 0.79, suggesting adequate interitem relatedness without excessive redundancy. The ICC value for the total score was 0.68 (95% CI 0.37-0.86; n=21), indicating moderate test-retest reliability.

## Discussion

### Key Findings and Interpretation

The newly developed PASQ assesses patients’ perceptions of the support for their personal agency. Although some participants required assistance to complete the survey, most were able to respond appropriately independently. This suggests that the PASQ is feasible for use in acute psychiatric wards. It also exhibited adequate convergent validity, acceptable divergent validity, good internal consistency, and moderate test-retest reliability among inpatients in acute psychiatric wards in Japan.

Although there are existing scales assessing support for similar concepts related to personal agency, such as empowerment and personal recovery [52-54], the PASQ is the first scale developed to evaluate support for personal agency. Several items generated from our study shared similarities with existing tools, such as the Health Care Climate Questionnaire, which assesses professionals’ support for patients’ autonomy [55]. Given that the Health Care Climate Questionnaire does not specifically assess care for patients with mental illness or acute care, the similarities observed between these tools suggest that there are fundamental elements involved in supporting patient personal agency, regardless of the type or stage of illness. However, each item of the PASQ reflects fundamental support elements in acute psychiatric wards. For example, the items related to a feeling of safety and respect reflect the importance of relational safety in acute psychiatric care, as highlighted in previous studies [56-58]. Support aligned with personal values, as captured by several items, contributes to self-understanding and a sense of consistency [59]. Furthermore, providing appropriate information and stage-appropriate choices, as addressed by other items, supports the regaining of control [8].

The relatively high scores for items related to a sense of safety and respect suggest that the psychiatric facilities prioritize these aspects during the acute phase of care. However, comparatively lower scores for items “Medical staff try to understand the reason for my actions,” “Treatment and care are in line with what I want and the way I wish to be,” and “Medical staff share future treatment plans and expectations with me” may reflect the challenge in supporting patients to participate in their treatment during the acute phase, despite its importance for personal agency [8,59,60].

PASQ and Brief INSPIRE-J scores exhibited a significant and strong correlation, confirming convergent validity. This finding supports the proposition that personal agency is key to promoting personal recovery [26-28]. The weak but significant negative correlation between PASQ and K6 did not support our hypothesis. However, this finding is consistent with previous studies, which found a negative relationship between personal agency and psychological distress [61,62]. It is therefore possible that perceived support for personal agency may be slightly associated with the extent of anxiety or depression, supporting the distinction between these concepts and the discriminant validity of the PASQ.

The PASQ exhibited high internal consistency, indicating sufficient homogeneity. Cronbach α values remained stable when items were deleted, suggesting limited redundancy. Item-to-total correlations were moderate to high, confirming that each item contributed meaningfully to the total score and that item deletion was not warranted. However, the moderate test-retest reliability suggests that the PASQ may not be entirely stable over time. This could be attributed to the rapid fluctuations in patients’ conditions in acute psychiatric settings [63] during the 2-week interval or the slightly smaller sample size in this study.

### Limitations and Strengths

This study has 2 main limitations. First, it was conducted at only 2 psychiatric hospitals, both of which provide relatively high-quality care. This may explain the relatively high scores observed, suggesting that our findings may not be generalizable to a broader range of clinical settings. Differences in staffing levels, staff training and attitudes, collaboration with community services, and the physical environment may all affect how support for personal agency is provided and perceived. Future research should therefore examine the applicability and validity of the PASQ in more diverse psychiatric settings. Second, the test-retest reliability was not excellent, with wide 95% CIs, which may reflect changes in patient symptoms and the care context during the 2-week interval; a shorter interval with a larger sample size might be more appropriate for acute psychiatric settings. Despite these limitations, this study is valuable as the first to develop a questionnaire for assessing perceived support for personal agency in acute psychiatric wards. It is also strengthened by the involvement of individuals with lived experience of acute psychiatric hospitalization, who helped to ensure that the PASQ reflects users’ perspectives in acute settings.

### Implications for Nursing Practice

The PASQ offers a practical approach to enable medical staff to understand how patients perceive support for personal agency in acute psychiatric wards and to apply these perspectives to clinical practice. Nurses, who work most closely with patients, face the challenge of maintaining a balance between delivering treatment, ensuring safety, and supporting patients’ agency [64]. The PASQ may be helpful for nurses to gain an understanding of patients’ subjective experiences and guide individualized care. Future studies could explore the use of the PASQ in staff training, interprofessional education, collaborative care planning in acute settings, and routine clinical reflection to enhance therapeutic engagement and communication. Furthermore, although the PASQ was developed and validated in acute settings, it may also be relevant in other contexts where agency may be compromised, such as long-term psychiatric hospitalization or trauma-related experiences. Future research should explore its applicability in such settings.

### Conclusion

In this study, we developed and validated the PASQ, a patient-reported questionnaire that assesses perceived support for personal agency in acute psychiatric inpatients. The questionnaire demonstrated adequate convergent validity, acceptable divergent validity, good internal consistency, and moderate test-retest reliability. The PASQ shows promise as a valuable tool for both clinical practice in acute psychiatric wards and future clinical research.

## Supplementary material

10.2196/83366 Multimedia Appendix 1 Personal Agency Support Questionnaire (PASQ), Japanese version.

10.2196/83366 Checklist 1 STROBE and COSMIN checklists.

## Abbreviations

COSMIN: Consensus-Based Standards for the Selection of Health Measurement Instruments
ICC: intraclass correlation coefficient
K6: Kessler 6-item Psychological Distress Scale
PASQ: Personal Agency Support Questionnaire
STROBE: Strengthening the Reporting of Observational Studies in Epidemiology
