# Supplementary material for: Personal Agency Support Questionnaire in Acute Psychiatric Inpatients: Development and Instrument Validation Study
Source: Asian Pac Isl Nurs J. 2026 Feb 3;10:e83366. doi: 10.2196/83366 (PMC12867469; doi:10.2196/83366)
Supplement: Multimedia Appendix 1 [file apinj-v10-e83366-s001.pdf]

Personal Agency Support Questionnaire

Questionnaire form: Japanese version

今回の入院について、「入院時から今まで」医療者からどれくらい支援をされているか、あてはまる数字ひとつに〇をつけてください。
※医療者とは、この病院に勤務しており、今回の入院であなたに関わっている人たちをさします。
特定の人ではなく、全体についてご回答ください。

		そう感じない	あまりそう感じない	どちらでもない	ややそう感じる	そう感じる
1	医療者は私に人としての敬意を払っている	0	1	2	3	4
2	医療者は私が率直な思いを表現できるようサポートしている	0	1	2	3	4
3	医療者は私が安全を実感できるよう関わっている	0	1	2	3	4
4	医療者は私が困りごとに対処できるようサポートしている	0	1	2	3	4
5	医療者は私の行動の理由を知ろうとしている	0	1	2	3	4
6	医療者は私の長所や大切にしていることを尊重している	0	1	2	3	4
7	治療やケアは、私本来のやりたいことやこうありたいという思いに沿っている	0	1	2	3	4
8	医療者は治療やケアの目的を私が納得できるタイミングや方法で説明している	0	1	2	3	4
9	医療者は今後の計画や見通しを私と共有している	0	1	2	3	4
10	医療者は状態の回復にあわせ、私が選び、決めることが増えるようサポートしている	0	1	2	3	4
