# Supplementary material for: Personal Agency Support Questionnaire in Acute Psychiatric Inpatients: Development and Instrument Validation Study
Source: Asian Pac Isl Nurs J. 2026 Feb 3;10:e83366. doi: 10.2196/83366 (PMC12867469; doi:10.2196/83366)
Supplement: Checklist 1 [file apinj-v10-e83366-s002.pdf]

Table 1: Revised items for reporting studies on measurement properties of Patient-Reported Outcome Measures (PROMs): General Reporting recommendations relevant for all studies on measurement properties

**General Reporting recommendations relevant for all studies on measurement properties**			
Item	Item name	Item description	Page No
Report section: Title			
T1	Title	Identify the report as a study of one or more measurement properties of a specific PROM to measure a specified construct in a specified population.	P.1, Title
Report section: Abstract			
A1	Objectives	Provide the specific objective(s) of the research, specifying (1) the name (and version, if relevant), and construct(s) of the PROM, (2) the measurement properties being evaluated, and (3) relevant study characteristics.	P.1, Abstract, Objective
A2	Design	Specify (details of the) study design used to evaluate the measurement properties.	P.1, Abstract, Methods
A3	Methods	Specify the methods for evaluating each measurement property.	P.1, Abstract, Methods
A4	Results	Provide the main results for all measurement properties evaluated.	P.1, Abstract, Results
A5	Discussion/ Conclusions	Provide a brief statement of the implications of the findings in the context of existing evidence on the PROM.	P.1, Abstract, Conclusions
Report section: Introduction			
I1	PROM	Specify the name and, if relevant, the version, and construct(s) of the PROM.	P.2, Introduction
I2	Target population & context of use	Specify the target population and context of use that the PROM was designed for.	P.2, Introduction
I3	State of knowledge & Rationale	Provide a description of the current scientific knowledge (what is known and not known) regarding the measurement properties of the PROM. Explain why the new study is necessary. Provide citations for the original development paper(s).	P.2, Introduction
I4	Objectives	Provide the specific objective(s) of the research, specifying (1) the name (and version, if relevant) of the PROM, (2) the measurement properties being evaluated, and (3) relevant study sample characteristics.	P.2, Introduction
Report section: General Methods			
GM1	Study design	Specify (details of the) study design used to evaluate the measurement properties.	P.2, Methods, Overall Design
GM2	Participants	Specify how the study participants were selected. Specify the inclusion and exclusion criteria	P.3, Methods, Phase3, Setting and Participants

GM3	PROM details	Provide details about the original version of the PROM as well as of the PROM version being studied, specify the conceptual framework (reflective/formative model), details on the structure (the number of items and subscales), the language, response scale, recall period, direction of scoring, and scoring algorithm of the PROM. Specify how the PROM was administered (e.g., in what setting, mode of administration (e.g. paper, electronic) what instructions were given), including the country in which it is administered	P.3, Methods, Phase3, Data Collection Procedures, Measures

GM4	Additional data collection	Describe why and how other data was collected (e.g., construct and measurement properties of the comparator instruments, characteristics of groups being compared, and rationale for choosing groups), including mode of administration (e.g., paper, electronic).	P.3, Methods, Phase3, Data Collection Procedures, and P4. Measures
GM5	Time points procedures	Provide all time points of all measurements.	P.3, Methods, Phase3, Setting and Participants, and P.5. Statistical Analysis
GM6	Justification for sample size	Provide a rationale for the sample size for all measurement properties analyses (including subgroups).	P.3, Methods, Phase3, Setting and Participants
GM7	Statistical analyses	Describe the statistical analyses corresponding to all objectives (see measurement properties specific boxes). Describe the criteria for good measurement properties. Name the statistical package used and the version.	P.5, Methods, Phase3, Statistical Analysis
GM8	Missing data	Describe approaches for dealing with missing data.	P.6, Methods, Statistical analysis (no missing data)
GM9	Unplanned analysis	Specify analyses that were unplanned and their rationale.	N/A
Report section: General results			
GR1	Participant characteristics	Provide study participants' characteristics, specified per subgroup if applicable.	P.6, Results, Phase 3, Study Participants and Table1.
GR2	Sample size	Provide the total number of participants included in the study and the sample size for each analysis.	P.6, Results, Phase 3, Study Participants and p.10 Reliability
GR3	Missing data	Provide amount of (proportion or count) and reasons for missing data for each analysis for the PROM, and for any analyses of other outcome measurement instruments.	P.6, Results, Phase 3, Study Participants
GR4	Results	Describe the results corresponding to all objectives (see measurement properties specific boxes).	P.6-7, Results, Phase 3, Distribution of the Responses, Convergent and Divergent Validity,

			Reliability
Report section: Discussion/conclusions			
DC1	Measurement property evidence	Provide the main findings and if each measurement property is sufficient or insufficient and why.	P.7, Discussion, Key findings and Interpretation
DC2	Practical relevance	Discuss the practical relevance of the findings in terms of recommendations for (not) using the PROM.	P.7-8, Discussion, Implications for Nursing Practice
DC3	Strengths and limitations	Discuss strengths and limitations of each study. For example, discuss if there were any potential biases in the study that could have impacted the results.	P.7, Discussion, Limitations and Strength
DC 4	Generalizability	Discuss generalizability of the results. For example, discuss whether the results could be generalized to other populations given the sample studied.	P.7, Discussion, Limitations and Strength
DC5	Instrument changes	Discuss what modifications are needed to the existing PROM.	P.7, Discussion, Key findings and Interpretation
DC6	Future research	Describe new research questions or hypotheses generated from these findings, and provide/describe the research needed to answer those questions.	P.7-8, Discussion, Implications for Nursing Practice
DC7	Conclusions	Provide the overall conclusions for the use of the PROM.	P.8, Discussion, Conclusion
Report section: Other information			
O1	Conflict of interest	State any conflict of interest you may have related to the PROM. This may include any involvement in the development of the PROM or any commercial funding or profit.	P.8, Conflicts of Interest

Table 2: Revised items for reporting studies on measurement properties of Patient-Reported Outcome Measures (PROMs): Specific Reporting recommendations for studies

**Specific Reporting recommendations for studies on Content Validity**			
Item	Item name	Item description	Page No
**Content validity: Methods**			
CV1	Relevance	Specify if and how patients and/or professionals were asked whether the instructions, each of the items, response options, and the recall period were relevant for the construct(s), population, and context of use.	P.3, Methods, Phase1
CV2	Comprehensi-veness	Specify whether and how patients and/or professionals were asked whether all key concepts are included in the PROM.	P.3, Methods, Phase1
CV3	Comprehens-ibility	Specify whether and how the comprehensibility of the PROM instructions, items, response options, and recall period was evaluated by patients and/or professionals.	P.3, Methods, Phase1
**Content validity: Results**			
CV4	Relevance	Specify if the instructions, all items, response options, and recall period were considered relevant, by patients and/or professionals, to the construct, population, and context of use.	P.5, Results, Phase1.
CV5	Comprehensi-veness	Specify whether patients and/or professionals considered all key concepts to be included in the PROM.	P.5, Results, Phase1.
CV6	Comprehens-ibility	Specify whether patients understood the PROM instructions, items, response options, and recall period as intended and/or whether professionals considered the instructions, items, response options, were appropriately worded.	P.5, Results, Phase1.
**Specific Reporting recommendations for studies on Structural Validity**			
Item	Item name	Item description	Page No
**Structural validity: Methods**			
SV1	Rationale for approach	Provide a rationale for the approach (e.g., factor analysis, Item Response Theory (IRT)/Rasch analysis) used.	N/A<br>(Because the PASQ was developed as a short assessment questionnaire in a checklist manner, following previous studies, we did not perform factor analysis.)
SV2	Statistical analyses	<u>Exploratory (EFA) or confirmatory factor analyses (CFA)</u><br>Describe the tested model (e.g., number of factors, which items included in which factor), method of estimation, type of correlation matrix, and methods and criteria for good model fit.<br><br><u>IRT/Rasch analysis</u><br>Describe the type of IRT/Rasch model, method of estimation, methods for checking assumptions (e.g., dimensionality, local independence, monotonicity), methods and criteria for good item parameters and model fit. Indicator software and version used.<br><br><u>Other approaches</u><br>Provide details of the methods if other approaches were used.	
**Structural validity: Results**			
SV3	Statistical analyses	<u>EFA or CFA</u><br>For EFA: provide all factor loadings, eigenvalues and % variance explained of the model reflecting the original PROM structure and best-fitting model if applicable.	N/A

		For CFA: provide all factor loadings and results for model fit indices of the model reflecting the original PROM structure and best-fitting model, if applicable.<br><br><u>IRT/Rasch analyses</u><br>Provide item/model fit results, all item parameters, and figures if appropriate (e.g., item characteristic curves, person-item mapping, item and/or test information functions).<br><br><u>Other approaches</u><br>Provide relevant results for other approaches used.	
**Specific Reporting recommendations for studies on Internal Consistency**			
Item	Item name	Item description	Page No
**Internal Consistency: Methods**			
IC1	Statistical analyses	Provide evidence for the unidimensionality of the PROM (subscales) and provide evidence of lack of local item dependence. Describe statistical methods used to calculate internal consistency.	P.5, Methods, Phase3, Statistical Analysis
**Internal Consistency: Results**			
IC2	Statistical analyses	Provide internal consistency results for each unidimensional scale or subscale separately.	P.7, Results, Phase3, Reliability and Table2.
**Specific Reporting recommendations for studies on Cross-Cultural Validity\Measurement Invariance**			
Item	Item name	Item description	Page No
**Cross-Cultural Validity\Measurement Invariance: Methods**			
CCV 1	Group variable	Describe the variable that differs between the subgroups that are being compared.	N/A
CCV 2	Rationale for approach	Provide a rationale for the approach (e.g., multi-group confirmatory<br>factor analysis, or logistic regression analysis of differential item function using Theta or sum scores, or other) used.	N/A
CCV 3	Statistical analyses	<u>Multiple-group confirmatory factor analysis (MGCFA)</u><br>Provide a description of the tested model, method of estimation, type of correlation matrix, and methods and criteria for change in model fit.<br><br><u>Differential Item Functioning (DIF) analyses</u><br>Provide the statistical approach used to calculate the patient's score (e.g., IRT/Rasch-based theta score, or (un)weighted sum score), a description of the tested regression models, and criteria used to flag items for DIF.<br><br><u>Other approaches</u><br>Provide details of the methods if other approaches were used.	N/A

**Cross-Cultural Validity\Measurement Invariance: Results**			
CCV 4	Statistical analyses	Provide results on changes or differences in model fit between tested models (e.g., MGCFA, IRT/Rasch analyses, logistic regression DIF analyses, or other approaches).	N/A

**Specific Reporting recommendations for studies on Reliability**			
Item	Item name	Item description	Page No
**Reliability: Methods**			
R1	Stability of patients	Provide arguments for assuming stability of patients on the construct of interest between the repeated measurements.	P.5, Methods, Phase3, Statistical Analysis
R2	Statistical analyses	Provide the specific intraclass correlation coefficient (ICC) or kappa formula used and justify the choice of the formula used.	P.5, Methods, Phase3, Statistical Analysis
R3	Sources of variation	Specify which source of variation was purposely varied across the repeated measurements.	P.5, Methods, Phase3, Statistical Analysis
R4	Time interval	Provide arguments for the appropriateness of the time interval.	P.5, Methods, Phase3, Statistical Analysis
**Reliability: Results**			
R5	Stability of patients	Describe whether patients were stable on the construct of interest between the repeated measurements.	P.7, Results, Phase3, Reliability and Discussion, Key findings and Interpretation, and Limitations and Strength
R6	Statistical analyses	Provide results for calculated statistics (ICC or kappa) and measures of precision (standard errors or confidence intervals). Provide results of all variance components, or contingency tables.	P.7, Results, Phase3, Reliability

**Specific Reporting recommendations for studies on Measurement Error**			
Item	Item name	Item description	Page No
**Measurement Error: Methods**			
ME1	Stability of patients	Provide arguments for assuming stability of patients on the construct of interest between the repeated measurements.	N/A
ME2	Statistical analyses	Provide and justify the specific statistical formula used for standard error of measurement (SEM), smallest detectable change (SDC), limits of agreement (LoA), or percentage (specific) agreement.	N/A
ME3	Sources of variation	Specify which source of variation was purposely varied across the repeated measurements.	N/A
ME4	Time interval	Provide arguments on the appropriateness of the time interval.	N/A
**Measurement Error: Results**			

ME5	Stability of patients	Describe whether patients were stable on the construct of interest between the repeated measurements.	N/A
ME6	Statistical analyses	Provide results for calculated statistics and measures of precision (if applicable). Provide results of all variance components included in the SEM, provide the systematic change or difference between the repeated measurements (when LoA was applied), or contingency tables.	N/A

Specific Reporting recommendations for studies on Criterion Validity			
Item	Item name	Item description	Page No
CriV1	Criterion	Justify the assumption that the comparator instrument is a reasonable gold standard.	N/A
**Criterion Validity: Methods**			
CriV2	Statistical analyses	Provide and justify the statistics used: correlations when criterion has continuous scores or area under the ROC curve, and sensitivity and specificity when criterion is dichotomous.	N/A
**Criterion Validity: Results**			
CriV3	Statistical analyses	Provide results for calculated statistics.	N/A

Specific Reporting recommendations for studies on Hypotheses Testing for Construct Validity			
Item	Item name	Item description	Page No
**Hypotheses Testing for Construct Validity: Methods**			
ConV1	Hypotheses	State hypotheses, and provide the rationale for each hypothesis.	P.5, Methods, Phase3, Statistical Analysis
ConV2	Statistical analyses	Specify all statistical methods used to test the hypotheses.	P.5, Methods, Phase3, Statistical Analysis
**Hypotheses Testing for Construct Validity: Results**			
ConV3	Statistical analyses	Provide all results and specify if each result is in accordance with its hypothesis.	P.7, Results, Phase3, Convergent and Divergent Validity, and Discussion, Key Findings and Interpretation

Specific Reporting recommendations for studies on Responsiveness			
Item	Item name	Item description	Page No
**Responsiveness: Methods**			
Resp1	Hypotheses	State the hypotheses, and provide the rationale for each hypothesis.	N/A
Resp2	Intervention/Exposure	Provide the intervention given or exposure in the interim period (or report that no intervention was provided).	N/A
Resp3	Statistical analyses	Specify all statistical methods used to test the hypotheses.	N/A
**Responsiveness: Results**			
Resp4	Statistical analyses	Provide results for calculated statistics, and specify, for the construct approach, if each result is in accordance with its hypothesis.	N/A

STROBE Statement—checklist of items that should be included in reports of observational studies

	Item No	Recommendation	Page No
**Title and abstract**	1	(*a*) Indicate the study's design with a commonly used term in the title or the abstract	P.1, Title & Abstract
		(*b*) Provide in the abstract an informative and balanced summary of what was done and what was found	P.1, Abstract
**Introduction**			
Background/ rationale	2	Explain the scientific background and rationale for the investigation being reported	PP.1-2, Introduction
Objectives	3	State specific objectives, including any prespecified hypotheses	P.2, Introduction
**Methods**			
Study design	4	Present key elements of study design early in the paper	P.2, Methods, Overall Design
Setting	5	Describe the setting, locations, and relevant dates, including periods of recruitment, exposure, follow-up, and data collection	P.3, Methods, Phase3, Setting and Participants
Participants	6	(*a*) *Cohort study*—Give the eligibility criteria, and the sources and methods of selection of participants. Describe methods of follow-up<br>*Case-control study*—Give the eligibility criteria, and the sources and methods of case ascertainment and control selection. Give the rationale for the choice of cases and controls<br>*Cross-sectional study*—Give the eligibility criteria, and the sources and methods of selection of participants	P.3, Methods, Phase3, Setting and Participants
		(*b*) *Cohort study*—For matched studies, give matching criteria and number of exposed and unexposed<br>*Case-control study*—For matched studies, give matching criteria and the number of controls per case	N/A
Variables	7	Clearly define all outcomes, exposures, predictors, potential confounders, and effect modifiers. Give diagnostic criteria, if applicable	PP.3-5, Methods, Phase3, Measures
Data sources/ measurement	8*	For each variable of interest, give sources of data and details of methods of assessment (measurement). Describe comparability of assessment methods if there is more than one group	P.3, Methods, Phase3, Data collection Procedures
Bias	9	Describe any efforts to address potential sources of bias	P.3, Methods, Phase3, Setting and Participants, and Data collection Procedures
Study size	10	Explain how the study size was arrived at	P.3, Methods, Phase3, Setting and Participants
Quantitative variables	11	Explain how quantitative variables were handled in the analyses. If applicable, describe which groupings were chosen and why	P.5, Methods, Phase3, Statistical Analysis
Statistical methods	12	(*a*) Describe all statistical methods, including those used to control for confounding	P.5, Methods, Phase3, Statistical

			Analysis
		(*b*) Describe any methods used to examine subgroups and interactions	N/A
		(*c*) Explain how missing data were addressed	P.6, Methods, Statistical analysis (no missing data occurred)
		(*d*) *Cohort study*—If applicable, explain how loss to follow-up was addressed<br>*Case-control study*—If applicable, explain how matching of cases and controls was addressed<br>*Cross-sectional study*—If applicable, describe analytical methods taking account of sampling strategy	N/A
		(*e*) Describe any sensitivity analyses	N/A
Results			
Participants	13*	(a) Report numbers of individuals at each stage of study—eg numbers potentially eligible, examined for eligibility, confirmed eligible, included in the study, completing follow-up, and analysed	P.6, Results, Phase3, Study Participants, Table1, and P.7 Reliability
		(b) Give reasons for non-participation at each stage	P.6, Results, Phase3, Study Participants
		(c) Consider use of a flow diagram	N/A (flow of participants described in text)
Descriptive data	14*	(a) Give characteristics of study participants (eg demographic, clinical, social) and information on exposures and potential confounders	P.6, Results, Phase3, Study Participants, and Table1
		(b) Indicate number of participants with missing data for each variable of interest	P.6, Results, Phase3, Study Participants, and Table1
		(c) *Cohort study*—Summarise follow-up time (eg, average and total amount)	N/A
Outcome data	15*	*Cohort study*—Report numbers of outcome events or summary measures over time	N/A
		Case-control study—Report numbers in each exposure category, or summary measures of exposure	N/A
		Cross-sectional study—Report numbers of outcome events or summary measures	P.6, Results, Phase3, Distribution of the Responses, and Figure 1
Main results	16	(*a*) Give unadjusted estimates and, if applicable, confounder-adjusted estimates and their precision (eg, 95% confidence interval). Make clear which confounders were adjusted for and why they were included	P.6, Results, Phase3, Convergent and Divergent Validity, and Reliability
		(*b*) Report category boundaries when continuous variables were categorized	N/A
		(*c*) If relevant, consider translating estimates of relative risk into absolute risk for a meaningful time period	N/A

Other analyses	17	Report other analyses done—eg analyses of subgroups and interactions, and sensitivity analyses	N/A
**Discussion**			
Key results	18	Summarise key results with reference to study objectives	P.7, Discussion, Key Findings and Interpretation
Limitations	19	Discuss limitations of the study, taking into account sources of potential bias or imprecision. Discuss both direction and magnitude of any potential bias	P.7, Discussion, Limitations and Strength
Interpretation	20	Give a cautious overall interpretation of results considering objectives, limitations, multiplicity of analyses, results from similar studies, and other relevant evidence	PP.7-8, Discussion, Key Findings and Interpretation, and Conclusion
Generalisability	21	Discuss the generalisability (external validity) of the study results	P.7, Discussion, Limitations and Strength
**Other information**			
Funding	22	Give the source of funding and the role of the funders for the present study and, if applicable, for the original study on which the present article is based	P.8, Funding

*Give information separately for cases and controls in case-control studies and, if applicable, for exposed and unexposed groups in cohort and cross-sectional studies.

Note: An Explanation and Elaboration article discusses each checklist item and gives methodological background and published examples of transparent reporting. The STROBE checklist is best used in conjunction with this article (freely available on the Web sites of PLoS Medicine at http://www.plosmedicine.org/, Annals of Internal Medicine at http://www.annals.org/, and Epidemiology at http://www.epidem.com/). Information on the STROBE Initiative is available at www.strobe-statement.org.
